# Expression and Clinical Significance of ILF2 in Gastric Cancer

**DOI:** 10.1155/2017/4387081

**Published:** 2017-07-31

**Authors:** Zi-huan Yin, Xing-wang Jiang, Wu-bing Shi, Qian-le Gui, Dong-feng Yu

**Affiliations:** ^1^Department of General Surgery, The First Affiliated Hospital of Anhui Medical University, Hefei, China; ^2^Department of Cardiology, Tianjin People's Hospital, Tianjin, China; ^3^Department of Hematology, The Second Affiliated Hospital of Anhui Medical University, Hefei, China

## Abstract

The aim of this study is to investigate the expression levels and clinical significance of ILF2 in gastric cancer. The mRNA and protein expression levels of ILF2 were, respectively, examined by quantitative real-time PCR (qRT-PCR) and Western blot from 21 paired fresh frozen GC tissues and corresponding normal gastric tissues. In order to analyze the expression pattern of ILF2 in GC, 60 paired paraffin-embedded GC slides and corresponding normal gastric slides were detected by immunohistochemistry (IHC) assay. The correlation between ILF2 protein expression levels and clinicopathological parameters, overall survival (OS), disease-free survival (DFS), and clinical prognosis were analyzed by statistical methods. Significantly higher levels of ILF2 were detected in GC tissues compared with normal controls at both mRNA and protein level. High expression of ILF2 was tightly correlated with depth of invasion, lymph node metastasis, pathological stage, and histological differentiation. Log-rank test showed that high expression of ILF2 was positively associated with poor clinical prognosis. Multivariate analysis identified that ILF2 was an independent prognostic factor for OS and DFS. Our findings suggest that ILF2 may be a valuable biomarker and a novel potential prognosis predictor for GC patients.

## 1. Introduction

Gastric cancer (GC) is one of the most common digestive tract cancers, and nearly one million new cases occur every year around the world [1]. The largest proportion of gastric cancer patients is mainly distributed in Eastern Asian countries especially in China and Japan. Almost 679.1 thousand new cases and 498 thousand deaths occur in China every year, over half of all incidences and deaths in the world. The morbidity and mortality of GC make it rank second place in all malignant tumors in China [1–3].

Currently, surgical treatment is regarded as a preferred procedure to cure GC [4]. A 5-year survival rate can be achieved in approximately 95% after surgery in early GC [5]. Although early gastric cancer can be curable, many cases are asymptomatic till advanced stages in which current effective therapeutic strategies are far from sufficient and optimal. In addition, it has been evidenced that a postoperative 5-year survival rate is still only about 30% in advanced GC [4, 6]. Therefore, new strategies to develop a new biomarker and effective therapeutic targets to improve poor prognosis need to be explored.

With the rapid development of molecular medicine, many biomarkers have been discovered to be associated with carcinogenesis, progression, and prognosis of GC. For example, many researchers observed that HER2 was overexpressed and related to poor prognosis in GC patients. The biotherapy of trastuzumab, which can specifically target HER2-positive GC, obtained a perfect curative effect [7, 8]. Besides, tumor markers CEA (carcinoembryonic antigen), CA19-9 (carbohydrate antigen 19-9), and CA125 (carbohydrate antigen 125) have been widely used to predict prognosis of GC patients in recent years [9]. Now, more and more researchers are engaged in finding new biomarkers that present higher sensitivity and specificity than current ones.

Interleukin enhancer-binding factor 2 (ILF2), which also known as nuclear factor 45 (NF-45), a subunit of NF-AT (nuclear factor of activated T cells), is encoded by a gene located on human chromosome 1 (1q21.3) [10]. It can be expressed in normal tissues such as testis, brain, and kidney and is primarily distributed in the nucleus [11]. ILF2 is a transcription factor that interacts with ILF3 (NF90) to regulate the expression of interleukin-2 gene and HS4-dependent interleukin-13 gene. ILF2 regulates gene expression at multiple levels including RNA transcription, processing, and translation [12–16]. Furthermore, ILF2 is involved in the replication process of several types of RNA viruses including hepatitis B virus and infectious bursal disease virus (IBDV) [17–19]. Recently, a great deal of studies have indicated that high expression of ILF2, which was observed in lymphoma, leukemia, glioma, cervical cancer, non-small-cell lung cancer, hepatocellular carcinoma, and esophageal squamous cell cancer, was significantly related to the poor prognosis of these malignant tumors [11, 20–26]. Although the functions of ILF2 have been extensively investigated, the biological functions and the molecular mechanisms of ILF2 in GC are not fully understood.

The mRNA and protein expression of ILF2 in gastric cancer were examined for the first time in this research. The possible relationship between high expression of ILF2 and clinicopathological parameters was further analyzed by statistical methods. Then, survival analysis was calculated to evaluate the prognostic value of ILF2 in GC. Finally, our data imply that ILF2 may play a pivotal role in the clinical prognosis of GC.

## 2. Materials and Methods

### 2.1. Patients and Tissue Specimens

In this retrospective study, a total of 60 paired formalin-fixed, paraffin-embedded GC specimens and corresponding normal gastric tissues were recruited from patients who were diagnosed with primary GC and have undergone partial or total gastrectomy at the Department of General Surgery in the First Affiliated Hospital of Anhui Medical University (Hefei, China) from December 2010 to January 2011. All patients, enrolled in our study, had no history of preoperative radiotherapy or chemotherapy. The main clinicopathological parameters of the patients were listed in Table 1. The cutoff levels, respectively, were 5.0 ng/ml, 37.0 U/ml, and 20.0 U/ml for preoperative serum CEA, CA19-9, and CA125 (within 1 week prior to gastrectomy). A result was considered positive when the serum level was higher than the cutoff level. The patients had a periodic follow-up every 3 months for the first 2 years after surgery and every 6 months for the next 3 years. The follow-up was ended until their death or January 2016. Local recurrence or metastasis of primary GC was confirmed by the levels of tumor markers including CEA, CA19-9, and CA125 and the inspection of B-type ultrasonic or computed tomography (CT) or magnetic resonance imaging (MRI) after gastrectomy. The time from the day of surgery to the day of death or tumor recurrence was defined as overall survival (OS) and disease-free survival (DFS). To assess the expression levels of ILF2 protein and ILF2 mRNA in GC tissues and normal gastric tissues, 21 paired fresh GC samples and corresponding adjacent normal tissues (at least 5 cm distant from the tumor edge), which were immediately frozen in liquid nitrogen after surgical removal and then were stored at −80°C, were used to extract protein and RNA for Western blot assay and qRT-PCR assay. The specimens used for the present research were obtained with patients' informed consent and were examined pathologically using the seventh edition of the tumor node metastasis (TNM) classification of the International Union against Cancer (UICC) criterion [27]. The research protocols were approved by the Ethics Committee of the First Affiliated Hospital of Anhui Medical University.

### 2.2. RNA Extraction, Reverse Transcription, and Quantitative Real-Time PCR

Total RNA was extracted from 21 paired fresh specimens, and corresponding normal specimens with TRIzol reagent (Invitrogen, USA) and RevertAid First Strand cDNA Synthesis Kit (Thermo Scientific, USA) were used for reverse transcription according to the manufacturer's protocol. Quantification of ILF2 mRNA was detected by quantitative reverse transcriptase PCR using Perfectstart SYBR Green qPCR Master Mix (Omega Bio-Tek, USA). The primers used for amplifying ILF2 are as follows: ILF2, forward primer: 5′-CGCCTCTTCAGTTGTCTGC-3′ and reverse primer: 5′-GACCACGGCCTCTGTCAC-3′; and glyceraldehyde-3-phosphate dehydrogenase (GAPDH), an internal control, forward primer: 5′-AGCCACATCGCTCAGACAC-3′ and reverse primer: 5′-GCCCAATACGACCAAATCC-3′. The amplification protocol was done with the following steps: denaturation at 95°C for 10 min, followed by 40 cycles of degeneration at 95°C for 15 s, annealing at 60°C for 20 s, and extension at 72°C for 40 s. The reaction was performed on the Stratagene Mx3000p Sequence Detection System (Applied Agilent, USA). All assays were done in triplicates. The 2^−ΔΔCt^ method was used to quantify the relative expression levels of ILF2 mRNA of each specimen.

### 2.3. Protein Extraction and Western Blot

Total proteins were extracted from 21 paired fresh frozen GC specimens and corresponding adjacent normal gastric specimens using RIPA lysis buffer and PMSF (Beyotime, China). The protein concentration was detected by BCA protein assay kit (Beyotime, China). Subsequently, an equivalent amount of protein of each paired specimen was separated by SDS-PAGE on 10% polyacrylamide gels and then was electrotransferred to 0.45 mm polyvinylidene fluoride membranes (Millipore, USA) for 1 h at 200 ma. After blocking in 5% nonfat milk diluted with TBST (tris-buffered saline/Tween-20) for 1 h at room temperature, the membranes were separately incubated with rabbit anti-ILF2 monoclonal antibody (1 : 3000; Abcam) and rabbit anti-GAPDH antibody (1 : 3000; Bioss) at 4°C overnight. On the second day, after washing 3 times with TBST per 10 min, the membranes were incubated with peroxidase-conjugated AffiniPure goat anti-rabbit IgG (1 : 6000; zsgb) for 1 h at room temperature. Finally, after washing 3 times with TBST per 10 min, the targeted protein was detected with the enhanced chemiluminescence system according to the manufacture's instruction. The intensity of ILF2 protein band was quantified by ImageJ software and normalized with GAPDH.

### 2.4. Immunohistochemistry Assay

ILF2 protein expression was measured by IHC staining in 4 *μ*m, formalin-fixed, paraffin-embedded tissue slides from 60 paired GC specimens and corresponding adjacent normal gastric specimens. Firstly, the tissue slides were deparaffined in xylene and rehydrated in different concentration gradients of ethyl alcohol. After washing 3 times with phosphate-buffered saline (PBS), the slides underwent antigen retrieval in citrate buffer (0.01 M, pH 6.0) in the microwave oven for 20 min. 3% hydrogen peroxidase (H_2_O_2_) was used to quench the endogenous peroxidase activity of the slides for 5 min. Then, the slides were incubated with the primary antibody of anti-ILF2 (1 : 200, Abcam) at 4°C overnight followed by washing 3 times per 5 min. PBS took the place of the primary antibody as the negative control. Polink-1 HRP DAB Detection System (ZSGB-BIO) was used to perform IHC staining as the manufacturer's instruction on the next day. All the results were, respectively, evaluated at high-magnitude microscope by two pathologists who had no knowledge about the clinicopathological parameters of the GC patients. The score of IHC staining was determined by staining intensity multiplied by positive cell percentage. According to staining intensity, four grades were divided as follows: a score of 0 for negative staining, 1 for weak staining (light yellow), 2 for moderate staining (claybank), and 3 for strong staining (sepia). As to the percentage of positive cell, four groups were classified: 3 points for >75%, 2 points for 25–75%, 1 point for <25%, and 0 points for no cells stained. Five fields were randomly selected, and the average score of the five fields was the final IHC staining score. At last, on the basis of IHC staining score, the expression level of ILF2 protein was divided into two groups: high expression (>2 scores) and low expression (≤2 scores).

### 2.5. Statistical Analysis

All statistical analyses were performed with the SPSS 17.0 software (SPSS, Chicago, IL, United States). The Pearson *X*^2^ test was devoted to evaluate the relationship between clinicopathological parameters and the expression level of ILF2 protein. The Kaplan-Meier method was used to calculate OS and DFS. The log-rank test was applied to compare the significant difference between groups. The Cox proportional hazard regression model was applied to univariate and multivariate analyses for predicting prognostic indicators. All *p* values were two-sided, and *p* < 0.05 was considered significantly different in statistics.

## 3. Results

### 3.1. Expression of ILF2 mRNA and Protein in Fresh GC Tissues

**Figure 1:**
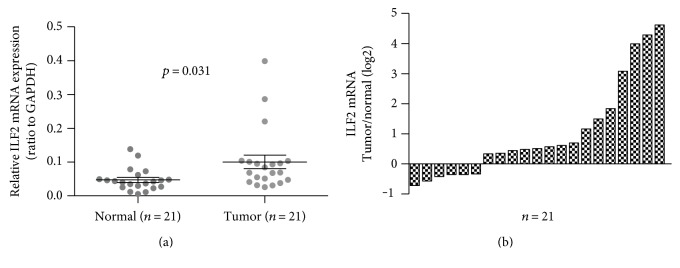
The expression levels of ILF2 mRNA in 21 paired samples by qRT-PCR. Scatter plots of the relative expression of ILF2 between GC tissues (tumor) and normal controls (normal) to GAPDH, GAPDH as an endogenous control (*p* = 0.031, paired *t*-test, two-tailed) (a). Bar plots of the relative expression of ILF2 in GC tissues compared with adjacent normal tissues; each patient was presented as the log2 ratio of tumor tissues/normal tissues (b).

**Figure 2:**
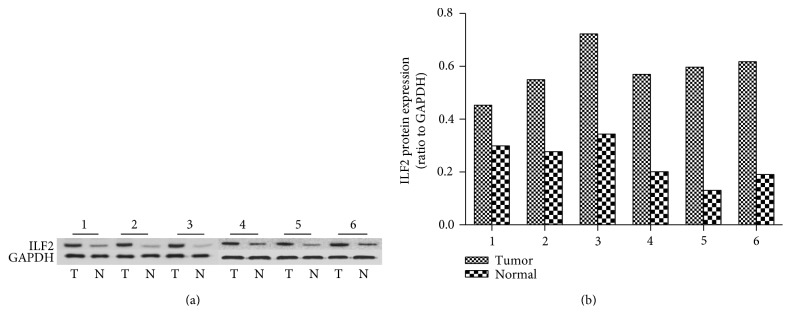
The expression levels of ILF2 protein in six representative paired clinical samples by Western blot analysis. The levels of ILF2 protein were higher in GC tissues (T) compared with adjacent normal tissues (N), GAPDH as an endogenous control (a). Relative quantification results of the intensity of ILF2 bands of GC tissues (tumor) and normal controls (normal) to GAPDH were quantified by gray analysis (b).

**Figure 3:**
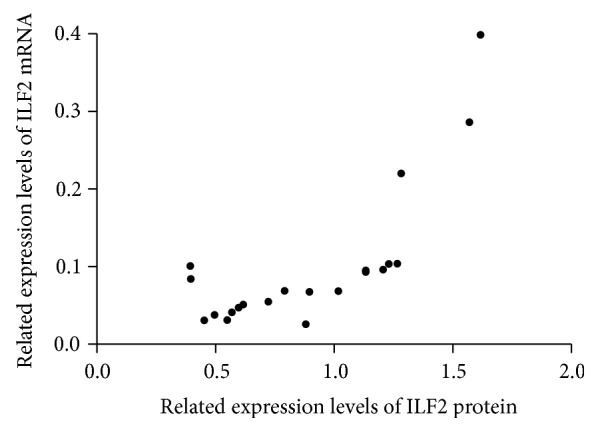
Correlation between ILF2 mRNA and ILF2 protein expression levels in 21 GC tissues was analyzed by Pearson correlation analysis (*r* = 0.753, *p* < 0.001).

As far as we know, high expression of ILF2 has been reported in several malignant tumors. However, the expression level of ILF2 in gastric cancer is not yet completely understood. In this study, qRT-PCR assay and Western blot assay were performed in 21 paired fresh GC tissues and corresponding adjacent normal gastric tissues. The expression levels of ILF2 mRNA in GC tissues were significantly higher than those in adjacent normal gastric tissues (Figure 1(a), *p* = 0.031, paired *t*-test). When defining <1-fold change as low expression and >1-fold change as high expression, it revealed that 71.43% (15/21) of GC tissues were high expression (Figure 1(b)). Western blot trial was performed in 21 paired samples to further confirm ILF2 protein expression. It was revealed that GC tissues exhibited a significantly higher expression of ILF2 protein compared with corresponding normal controls (*p* < 0.05, Wilcoxon matched-pairs signed-ranks test). The result of 6 paired representative samples was shown in Figure 2. From the above evidence, it is concluded that ILF2 is upregulated at both mRNA and protein levels in human GC. In addition, the positive correlation was observed between ILF2 mRNA and ILF2 protein expression levels (*r* = 0.753, *p* < 0.001, Figure 3).

### 3.2. Immunohistochemical Analysis of ILF2 in GC

**Figure 4:**
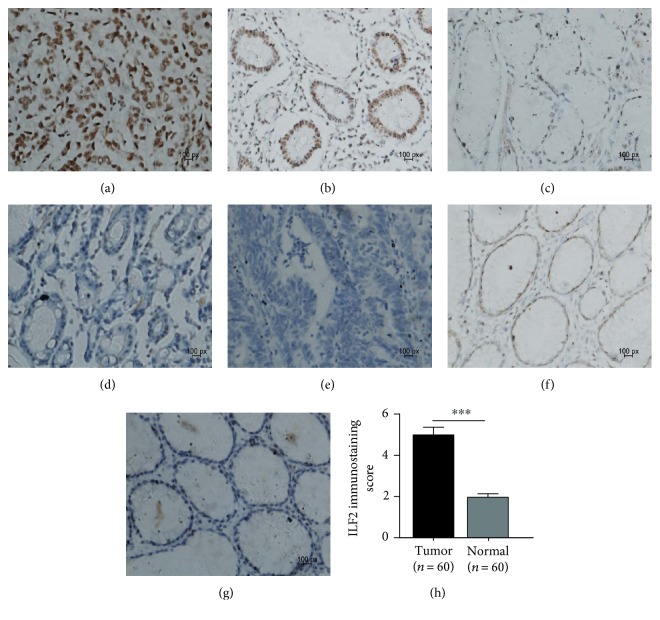
Representative microphotographs for ILF2 protein expression by IHC staining in GC tissues and corresponding normal gastric tissues. ILF2 protein in GC tissues in strong staining (sepia) (a), moderate staining (claybank) (b), weak staining (light yellow) (c), no staining (d), and negative controls (e). Weak staining (light yellow) (f) and barely stained (g) adjacent normal gastric tissues. The comparison of ILF2 immunostaining score between GC tissues (*n* = 60) and normal gastric tissues (*n* = 60); the results are presented as mean ± SEM (^∗∗∗^*p* < 0.001) Wilcoxon matched-pairs signed-ranks test (h) (original magnification: ×400 in (a)–(g)).

Our study illuminated that ILF2 mRNA and protein were overexpressed in GC. To evaluate the association of ILF2 protein expression levels with clinicopathological parameters, a further IHC staining experiment was conducted consisting of 120 tissues slides (60 GC and 60 normal controls). According to the IHC results, ILF2 protein was mainly expressed in the nucleus of GC cells (Figures 4(a), 4(b), and 4(c)). The overexpressed rate of ILF2 in GC tissues (61.67%, 37/60) was significantly higher than that in normal controls (20%, 12/60) (*p* < 0.001, Pearson *X*^2^ test). The mean score of ILF2 IHC staining in GC tissues (4.990 ± 0.378) was significantly higher than that in normal controls (1.967 ± 0.163) (*p* < 0.001, Wilcoxon matched-pairs signed-ranks test, Figure 4(h)).

### 3.3. Relationship between the Expression of ILF2 Protein and Clinicopathological Characteristics of GC Patients

The characteristics of the patients were listed in Table 1. Table 1 showed that the expression of ILF2 protein was significantly associated with histological differentiation (*p* < 0.001), depth of invasion (*p* = 0.046), lymph node metastasis (*p* = 0.009), and TNM stage (*p* < 0.001). The expression levels of ILF2 protein in patients with poorer differentiation, deeper invasion (T3/T4), lymph node metastasis, and TNM stage III/IV were significantly higher than those with well/moderate differentiation, T1/T2, no lymph node metastasis, and TNM stage I/II. No direct effects of gender, age, location, size, surgical resection, serum CA19-9, CEA, and CA125 were observed in ILF2 expression (*p* > 0.05).

### 3.4. Survival Analysis of ILF2 Expression

**Figure 5:**
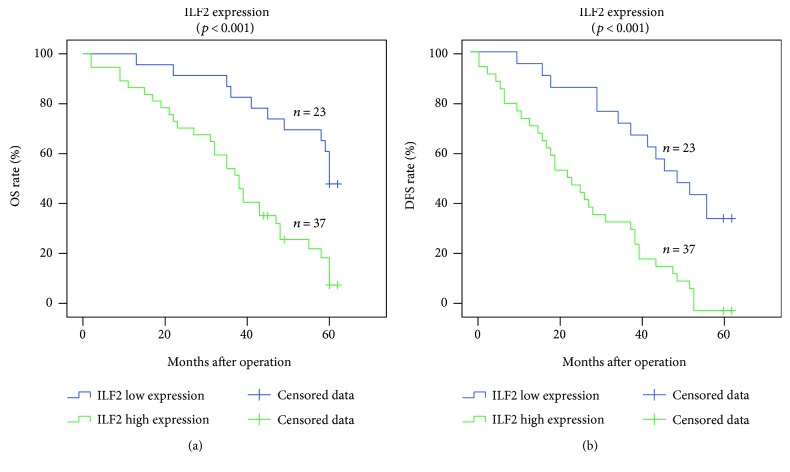
Kaplan-Meier survival analysis and log-rank test for OS and DFS of GC patients. The OS of GC patients with ILF2 high expression and low expression (a). The DFS of GC patients with ILF2 high expression and low expression (b).

In this retrospective study, postoperative follow-up was conducted: the median time was 45 months (range 2–62 months) and the mean time was 42.950 ± 2.398 (mean ± SE) months. Kaplan-Meier survival analysis was calculated to evaluate the prognostic values of ILF2 protein in GC patients. The median and mean survival times of GC patients with high ILF2 expression, respectively, were 38 months and 36.519 ± 2.985 months, which were shorter than those with low expression groups (median 60 months, mean 53.043 ± 2.916 months) (*p* < 0.001, log-rank test, Figure 5(a)). Similarly, the median and mean DFS times of GC patients with high ILF2 expression separately were 26 months and 28.243 ± 2.901 months, which were shorter than those of GC patients with low ILF2 expression (median 52 months, mean 47.087 ± 3.377 months) (*p* < 0.001, log-rank test, Figure 5(b)). In summary, high expression of ILF2 is significantly associated with poor clinical prognosis and increases the risk of disease recurrence in GC patients.

### 3.5. Univariate and Multivariate Cox Regression Analyses

To explore the independent prognostic factor for DFS and OS of GC patients, univariate and multivariate Cox regression analyses were performed in this study. Univariate Cox regression analysis suggested that ILF2 expression (*p* < 0.001), TNM stage (*p* = 0.001), histological differentiation (*p* = 0.026), depth of invasion (*p* = 0.007), CA19-9 (*p* = 0.009), CA125 (*p* = 0.007), and CEA (*p* = 0.022) were positive prognostic factors for DFS. Similarly, univariate Cox regression analysis indicated that ILF2 expression (*p* < 0.001), TNM stage (*p* < 0.001), histological differentiation (*p* = 0.017), depth of invasion (*p* = 0.010), CA19-9 (*p* = 0.012), CA125 (*p* = 0.003), and CEA (*p* = 0.021) also were positive prognostic factors for OS. Subsequently, multivariate Cox regression analysis was carried out to analyze these positive prognostic factors. It further supported that ILF2 expression (*p* < 0.001), depth of invasion (*p* = 0.002), CA19-9 (*p* = 0.002), and CA125 (*p* = 0.014) were independent prognostic factors for DFS of GC patients. In addition, ILF2 expression (*p* = 0.022), depth of invasion (*p* = 0.007), TNM stage (*p* = 0.005), CA19-9 (*p* = 0.004), and CA125 (*p* = 0.001) were independent prognostic factors for OS of GC patients (Table 2). In conclusion, ILF2 expression can be an independent prognostic factor for DFS and OS of GC patients.

## 4. Discussion

It is generally known that GC is one of the most common causes of cancer-related deaths around the world [28]. Despite the improvement of new diagnostic and therapeutic techniques, the 5-year survival rate remains about 30% [4, 6]. Therefore, more effective and specific methods are needed to enhance the survival rate. With the development of molecular biology techniques, researchers find that multiple genetic and epigenetic alterations are significantly correlated with genesis, progression, and prognosis of cancers [29, 30]. Genes encoding proteins that are expressed specifically in cancers and function in the oncogenetic process may be ideal diagnostic biomarkers or therapeutic targets [29]. For instance, the upregulation of HER2 provides us a signal that GC may be occurring. Furthermore, targeted therapies for HER2-positive GC patients have achieved a perfect curative effect [8]. In a word, if sufficient knowledge of gene expression changes that occur in the process of carcinogenesis is available, it can help us to improve the curative effect for cancers. The researches on the expression of genes and proteins that play an important role in cancers have become more and more popular in recent years.

ILF2 combines with ILF3 to participate in the process of mitosis, transcription regulation, DNA repair, microRNA processing, and virus replication. In addition, the role of ILF2 as a tumor promoter has also been recognized [11, 16, 17, 23, 31–33]. It was identified that the high expression of ILF2 was associated with poor outcome of ESCC patients and that ILF2 can promote the progression of ESCC via regulating cell cycle G0/G1-S transition [23]. Investigations have demonstrated that the altered regulation of cell cycle may lead to uncontrolled growth and contribute to oncogenesis [34]. The experiments confirmed that knockdown of ILF2 can delay G0/G1-S transition and abolish the proliferation effect of ESCC cells [23]. To the best of our knowledge, ILf2 also could inhibit the apoptosis of liver cancer cells through regulating the expression of B-cell lymphoma 2 (Bcl-2), Bcl-2 related ovarian killer (Bok), Bcl-2-associated X protein (BAX), and cellular inhibitor of apoptosis 1 (cIAP1) to facilitate the genesis and progression of liver cancer [20]. Furthermore, it was reported that ILF2 had similar functions in other malignant tumors such as gliomas and non-small-cell lung cancer [22, 24]. According to the above documents, ILF2 may promote the occurrence and progression of GC via accelerating cell cycle transition or inhibiting cell apoptosis. However, the detailed mechanisms of ILF2 in GC need to be further studied. Agents that can target ILF2 need to be vigorously developed to provide an efficient strategy to conquer GC.

It is worth noting that this is the first research to investigate the expression and clinical significance of ILF2 in GC. It was found that ILF2 was overexpressed at both mRNA and protein levels in GC tissues compared with corresponding normal controls. This result indicated that ILF2 might function as a tumor promoter in GC. Next, the relationship between ILF2-positive expression and clinicopathological characteristics of GC patients was assessed. The result revealed that the high expression of ILF2 was significantly related to histological differentiation, TNM stage, depth of invasion (T classification), and lymph node metastasis; moreover, the more advanced the tumor was, the higher possibility the overexpression of ILF2 existed. In short, ILF2 may play a vital role in the occurrence and progression of GC.

It had been reported in some previous literature that the high expression of ILF2 increased the risk of poor clinical prognosis in certain tumors [20, 22–24]. In this research, it was not a surprise that a similar outcome was found in GC. Kaplan-Meier survival analysis manifested that the high expression of ILF2 should take responsibility for shorter OS and DFS. These results indicated that ILF2 may relate to the poor clinical prognosis and recurrence of GC. Multivariate Cox regression analysis revealed that ILF2 was a negative and independent prognostic factor for both OS and DFS. These results are in line with previous researches which were conducted in other human malignancies such as pancreatic ductal adenocarcinoma and esophageal squamous cell carcinoma [24, 26]. Hence, it may be more clear to recognize that ILF2 may be a novel biomarker for predicting prognosis and recurrence of GC.

There are some limitations in this study. Firstly, although the expression and prognostic significance of ILF2 in GC have been evaluated, the specific functions and molecular mechanisms of ILF2 in GC need to be further investigated based on in vivo and in vitro experiments. Secondly, because of the source of tissue specimens, there is only a small number of tissue samples. Thus, a larger number of tissue samples are needed to exhibit the clinical significance of ILF2 in gastric cancer.

In summary, this study for the first time confirms that ILF2 is overexpressed and is an independent prognostic factor for OS and DFS of GC. ILF2 may be a potential novel prognostic indicator for GC. At the same time, it also provides a new idea for the treatment of GC.

## Figures and Tables

**Table 1:** Relationship between ILF2 expression and clinicopathological parameters (*n* = 60).

Parameters	ILF2 expression			
	High (*n* = 37)	Low (*n* = 23)	*X* ^2^	*p* value
Gender				
Male	21	13	0.000	0.986
Female	16	10		
Age (years)				
>60	19	14	0.519	0.471
≤60	18	9		
Location				
Cardia	19	12	0.004	0.951
Body/antrum	18	11		
Size (cm)				
>5	13	9	0.097	0.755
≤5	24	14		
Histological differentiation				
Well/moderate	7	15	13.092	0.000^∗^
Poor/not	30	8		
Depth of invasion (T classification)				
T1/T2	6	9	3.972	0.046^∗^
T3/T4	31	14		
Lymph node metastasis				
No	10	14	6.769	0.009^∗^
Yes	27	9		
Surgical resection				
Partial	10	7	0.081	0.776
Total	27	16		
TNM stage				
I/II	9	16	11.944	0.001^∗^
III/IV	28	7		
CA19-9				
Positive	13	6	0.537	0.464
Negative	24	17		
CEA				
Positive	13	7	0.141	0.707
Negative	24	16		
CA125				
Positive	12	6	0.272	0.602
Negative	25	17		

TNM: tumor node metastasis; ^∗^*p* < 0.05 and was defined as statistically significant.

**Table 2:** Univariate and multivariate Cox regression analyses for OS and DFS of GC patients.

Variables	OS		DFS	
	RR (95% CI)	*p* value	RR (95% CI)	*p* value
*Univariate analysis*				
Gender (male versus female)	1.856 (0.976–3.529)	0.059	1.579 (0.884–2822)	0.123
Age (years) (>60 versus ≤60)	1.799 (0.944–3.429)	0.074	1.701 (0.948–3.050)	0.075
Location (cardia versus body/antrum)	1.095 (0.589–2.035)	0.774	0.794 (0.448–1.406)	0.429
Size (cm) (>5 versus ≤5)	1.073 (0.560–2.056)	0.831	1.344 (0.748–2.416)	0.323
Histological differentiation (well/moderate versus poor/not)	2.403 (1.173–4.926)	0.017^∗^	2.040 (1.089–3.823)	0.026^∗^
Depth of invasion (T1/T2 versus T3/T4)	3.135 (1.311–7.495)	0.010^∗^	2.741 (1.319–5.695)	0.007^∗^
Lymph node metastasis (no versus yes)	1.698 (0.865–3.257)	0.111	1.578 (0.878–2.837)	0.128
TNM stage (I/II versus III/IV)	4.727 (2.218–10.074)	<0.001^∗^	2.897 (1.565–5.365)	0.001^∗^
ILF2 expression (high versus low)	4.496 (2.045–9.883)	<0.001^∗^	3.251 (1.699–6.224)	<0.001^∗^
CA19-9 (positive versus negative)	2.254 (1.191–4.264)	0.012^∗^	2.223 (1.219–4.054)	0.009^∗^
CA125 (positive versus negative)	2.677 (1.399–5.122)	0.003^∗^	2.340 (1.268–4.319)	0.007^∗^
CEA (positive versus negative)	2.133 (1.117–3.995)	0.021^∗^	2.103 (1.105–3.669)	0.022^∗^
*Multivariate analysis*				
TNM stage (I/II versus III/IV)	3.462 (1.451–8.260)	0.005^∗^	—	—
ILF2 expression (low versus high)	2.996 (1.173–7.654)	0.022^∗^	3.464 (1.727–6.947)	<0.001^∗^
Depth of invasion (T1/T2 versus T3/T4)	3.849 (1.452–10.206)	0.007^∗^	3.669 (1.613–8.343)	0.002^∗^
CA19-9 (positive versus negative)	2.911 (1.414–5.994)	0.004^∗^	3.210 (1.538–6.701)	0.002^∗^
CA125 (positive versus negative)	3.632 (1.695–7.786)	0.001^∗^	2.391 (1.193–4.791)	0.014^∗^

95% CI: 95% confidence interval; RR: relative risk; TNM: tumor node metastasis; ^∗^*p* < 0.05.
